# The potential for diversion of prescribed opioids among orthopaedic patients: Results of an anonymous patient survey

**DOI:** 10.1371/journal.pone.0256741

**Published:** 2021-08-26

**Authors:** Kala Sundararajan, Prabjit Ajrawat, Mayilee Canizares, J. Denise Power, Anthony V. Perruccio, Angela Sarro, Luis Montoya, Y. Raja Rampersaud, the University Health Network Division of Orthopaedic Surgery

**Affiliations:** 1 Division of Orthopaedic Surgery, Schroeder Arthritis Institute, University Health Network, Toronto, Ontario, Canada; 2 Krembil Research Institute, University Health Network, Toronto, Ontario, Canada; 3 Department of Surgery, Division of Orthopaedics, University of Toronto, Toronto, Ontario, Canada; 4 Institute of Health Policy, Management & Evaluation, Dalla Lana School of Public Health, University of Toronto, Toronto, Ontario, Canada; International Medical University, MALAYSIA

## Abstract

**Introduction:**

Diversion of prescription opioid medication is a contributor to the opioid epidemic. Safe handling practices can reduce the risk of diversion. We aimed to understand: 1) if orthopaedic patients received instructions on how to safely handle opioids, 2) their typical storage/disposal practices, and 3) their willingness to participate in an opioid disposal program (ODP).

**Methods:**

Cross-sectional study of adult orthopaedic patients who completed an anonymous survey on current or past prescription opioid use, instruction on handling, storage and disposal practices, presence of children in the household, and willingness to participate in an ODP. Frequencies and percentages of responses were computed, both overall and stratified by possession of unused opioids.

**Results:**

569 respondents who reported either current or past prescription opioid use were analyzed. 44% reported receiving storage instructions and 56% reported receiving disposal instructions from a health care provider. Many respondents indicated unsafe handling practices: possessing unused opioids (34%), using unsafe storage methods (90%), and using unsafe disposal methods (34%). Respondents with unused opioids were less likely to report receiving handling instructions or using safe handling methods, and 47% of this group reported having minors or young adults in the household. Respondents who received storage and disposal instructions were more likely to report safe storage and disposal methods. Seventy-four percent of respondents reported that they would participate in an ODP.

**Conclusion:**

While many orthopaedic patients report inadequate education on safe opioid handling and using unsafe handling practices, findings suggest targeted education is associated with better behaviours. However, patients are willing to safely dispose of unused medication if provided a convenient option. These findings suggest a need to address patient knowledge and behavior regarding opioid handling to reduce the risk of opioid diversion.

## Introduction

The opioid epidemic is a critical public health issue in Canada [1]. Opioid-related hospitalizations and rates of opioid poisoning emergency department (ED) visits continue to increase [2]. Rates of hospitalization for opioid poisoning are growing among youth (age 15 to 24) and younger adults (age 25 to 44), with respective increases of 53% and 62% from 2013 to 2017 [3]. In 2019, one in ten Ontario students in intermediate or secondary school reported using prescription opioids not prescribed to them, with the majority reporting that they obtained these drugs from their home [4]. However, opioid diversion in the household has received little attention from the medical community despite it being a significant source of opioid misuse in this vulnerable group.

Opioids are commonly prescribed for musculoskeletal pain and post-surgical pain [5–7]. Among physicians, orthopaedic surgeons are the third highest group of opioid prescribers [8,9]. Consequently, orthopaedic surgery patients may be important contributors to opioid diversion, as the majority of surgical patients use less than half of their prescribed opioid medication and often keep the surplus [8,9]. While current efforts to address over-prescription of opioids are a critical step towards reduction of opioid diversion [10,11], little is known about how opioid handling in the household may relate to diversion. The primary objectives of this study were to develop a better understanding of: 1) whether orthopaedic patients receive instruction on how to safely handle opioids, 2) their typical storage and disposal practices, and 3) their willingness to participate in a hypothetical hospital-based opioid disposal program. Secondarily, we examined how these factors varied among patients with and without unused opioids at home, and whether patient responses varied with the presence of children or young adults in the home.

## Materials and methods

### Study design

Cross-sectional study of consecutive patients who visited the outpatient orthopaedic clinic at Toronto Western Hospital from May to July 2017. Patients were invited to complete and return an anonymous paper survey to an unmanned box in the clinic. Respondents who reported current or former use of opioids were included in the analysis sample.

### Data collection

The survey collected respondents’ sex, age group, history of being prescribed opioids, previous instruction regarding safe opioid handling practices, actual handling practices, and the presence of children and/or young adults in their household (see S1 Appendix). Survey items were generated based on review of existing studies and Canadian recommendations [12,13].

### Survey measures

#### Opioid use

Respondents were asked if they had ever been prescribed opioid/narcotic pain medications (options: never; prescribed in the past but not currently using; currently using sometimes but not daily; currently using daily). This question was not limited to prescription by an orthopaedic surgeon.

#### Unused opioids at home

Respondents were asked if they had any opioids at home that were no longer being used or were expired (“Yes, prescribed to me”, “Yes, prescribed to someone else”, or “No”). Respondents reporting any unused opioids at home were collapsed into one group.

#### Minors and/or young adults in the home

Respondents were asked if children, teenagers, or young adults lived in or visited their home (“No”, “Yes, young children aged 0–6 years”, “Yes, older children aged 7–12 years”, “Yes, teenagers aged 13–17 years”, and/or “Yes, young adults aged 18–25 years”).

#### Instruction regarding safe opioid storage/disposal

Respondents who had been prescribed opioids were asked if they had ever received information on how to a) store and b) dispose of opioids (“Yes, from a pharmacist”, “Yes, from a healthcare provider”, “Yes, from another source”, “No, I have never received such information”, and/or “I don’t know”). Appropriate instruction was defined as receiving the information from a pharmacist or health care provider.

#### Opioid storage

Respondents were asked how they typically stored opioids in their home (“Cabinet/storage with a latch”, “Cabinet/storage with a lock”, “Cabinet/storage with no latch or lock”, or “Other”). Canadian guidelines state that opioids should be stored “out of sight and reach of children and pets” and “in a safe place to prevent theft, problematic use or accidental exposure” [12]. For this analysis, locked opioid storage locations were considered secure, in accordance with existing studies [14–17].

#### Opioid disposal

Respondents were asked how they typically disposed of opioids (“Flush down the toilet or sink”, “Throw away in garbage”, “Mix with undesirable material [e.g. kitty litter, coffee grounds] and throw in garbage”, “Return to a pharmacy or community take-back program”, “I would not dispose of it”, and/or “Other”). Safe opioid disposal was defined in accordance with Canadian guidelines^13^ as either a) returning unused opioids to a pharmacy or community take-back program, or b) mixing the medication with undesirable material and throwing it in the garbage.

#### Sharing opioid medication

Respondents were asked if they had ever shared opioids with another person (“Yes, I have shared opioid/narcotic medication prescribed to me”, “Yes, I have used opioid/narcotic medication prescribed to someone else”, and/or “No”).

#### Participation in a hospital-based take-back program

Respondents were asked if they would be willing to safely dispose of unused opioid medication (given that they had any) at their next hospital appointment (“Yes” or “No”). Respondents who were not willing to participate were asked to specify their reason(s), with options: “I am afraid my pain will come back and I’m not willing to be without it”, “If I need it again, I will not be able to get it from a doctor in a timely manner”, “I do not want to throw it away because I paid for it”, “My friends or family members may need it”, “I would prefer to dispose of it myself”, and/or “Other”.

### Statistical analysis

Responses were summarized with frequencies and percentages, both in the full sample and stratified by presence of unused opioid medication in the home. Respondents who reported any unused opioids at home were compared to respondents with no unused opioids using chi-square independence tests. Confidence intervals for differences in proportions were also calculated using the Agresti and Coull method [18], with Bonferroni adjustment to control family-wise error when necessary [19]. A descriptive comparison of reported opioid storage method versus presence of children, teenagers, and/or young adults in the home versus was also performed. After confirming that all survey items had a completion rate of at least 90%, missing responses were removed using pairwise deletion.

### Ethics approval

This study was approved by the University Health Network Research Ethics Board (approval number 19–5808) as a retrospective analysis of data collected anonymously for a clinical quality initiative. Since identifying information was not collected, the informed consent requirement was waived.

## Results

Of 784 total respondents, 569 who indicated that they had been prescribed opioid medication in the past were included in the analysis sample. The remaining respondents either reported that they had never been prescribed opioids (n = 182) or did not indicate their use of opioids (n = 33). Sixty percent of the sample were women, and 58% were age 55 or older (Table 1). Seventy-two percent were former opioid users and 28% were current users. Forty-two percent reported at least one child, teenager, or young adult living in or visiting their home. Overall, 34% of respondents reported having unused opioids at home. Having unused opioids at home was not significantly related to age group, gender, or having minors/young adults at home, but was more common among former versus current opioid users (p<0.001).

**Table 1:** Characteristics of opioid-using respondents, overall and by presence of unused opioids at home.

			Unused opioid medication at home			
Measure	Category	All opioid ever-users, % (count) N = 569	No unused opioids, % (count) N = 374	Any unused opioids, % (count) N = 195	Difference [95% CI]	P
**Age group^a^^,^^b^**	18–24	3.9% (22)	3.5% (13)	4.6% (9)	1.1% [-3.5, 6.2]	0.316
	25–34	11.1% (63)	10.2% (38)	12.8% (25)	2.6% [-4.8, 10.4]	
	35–44	10.7% (61)	11.8% (44)	8.7% (17)	-3.1% [-9.9, 4.1]	
	45–54	16.7% (95)	16.9% (63)	16.4% (32)	-0.5% [-9.0, 8.4]	
	55–64	27.8% (158)	29.5% (110)	24.6% (48)	-4.9% [-15.0, 5.5]	
	65–74	21.1% (120)	18.8% (70)	25.6% (50)	6.9% [-2.9, 16.8]	
	75+	8.6% (49)	9.4% (35)	7.2% (14)	-2.2% [-8.4, 4.4]	
	Age group not reported	— (1)	— (1)	— (0)		
**Female sex^a^**	—	59.6% (334)	58.9% (216)	61.1% (118)	2.3% [-6.3, 10.7]	0.665
	Sex not reported	— (9)	— (7)	— (2)		
**Prescription opioid medication use^b^**	Former	71.5% (407)	65.2% (244)	83.6% (163)	18.3% [10.0, 26.2]	<0.001*
	Sometimes	11.8% (67)	14.4% (54)	6.7% (13)	-7.8% [-13.3, -1.7]	
	Daily	16.7% (95)	20.3% (76)	9.7% (19)	-10.6% [-17.0, -3.6]	
**Do children, teenagers, or young adults live in your home or visit your home?^c^**	Young children (age 0–6)	12.3% (70)	10.7% (40)	15.4% (30)	4.7% [-1.2, 10.8]	0.138
	Older children (age 7–12)	13.5% (77)	14.4% (54)	11.8% (23)	-2.6% [-8.2, 3.4]	0.456
	Teenagers (age 13–17)	12.0% (68)	9.9% (37)	15.9% (31)	6.0% [0.2, 12.1]	0.050
	Young adults (age 18–25)	20.7% (118)	19.8% (74)	22.6% (44)	2.8% [-4.2, 10.0]	0.505
	None of the above	58.3% (332)	61.2% (229)	52.8% (103)	-8.4% [-16.9, 0.2]	0.066

CI = confidence interval.

* P < 0.05 (chi-square test of independence: No unused opioids versus any unused opioids).

^a^ Percentages calculated excluding missing responses.

^b^ Confidence intervals adjusted using Bonferroni correction to control familywise error.

^c^ Multiple selections were permitted; percentages may total more than 100%.

Fewer than half of respondents reported that they received appropriate instruction in opioid medication handling: 43% received storage instructions and 37% received disposal instructions from a pharmacist or other healthcare provider (HCP) (Table 2). Many respondents stated that they received no storage (37%) or disposal (43%) instructions. Compared to respondents who reported no unused opioids at home, those who did have unused opioids were significantly less likely to have received storage instructions (36% vs. 46%, p = 0.035) or disposal instructions (27% vs. 43%, p<0.001) from a pharmacist or other HCP.

**Table 2:** Opioid handling information sources and behaviours, overall and by presence of unused opioids at home.

			Unused opioid medication at home			
Measure	Category	All opioid ever-users, % (count) N = 569	No unused opioids, % (count) N = 374	Any unused opioids, % (count) N = 195	Difference [95% CI]	P
**Guidance on safe storage of opioids**						
**Opioid storage information source(s)^c^**	Pharmacist	35.5% (202)	37.7% (141)	31.3% (61)	-6.4% [-14.4, 1.8]	0.154
	Other healthcare provider	13.4% (76)	15.8% (59)	8.7% (17)	-7.1% [-12.3, -1.4]	0.026*
	Another source	3.2% (18)	4.0% (15)	1.5% (3)	-2.5% [-5.1, 0.6]	0.178
	I have never received this information	37.3% (212)	32.4% (121)	46.7% (91)	14.3% [5.8, 22.7]	0.001*
	I don’t recall	15.3% (87)	15.5% (58)	14.9% (29)	-0.6% [-6.7, 5.8]	0.938
**Received storage information from a pharmacist or other healthcare provider**	—	42.7% (243)	46.0% (172)	36.4% (71)	-9.6% [-17.9, -1.1]	0.035*
**Guidance on safe disposal of opioids**						
**Opioid disposal information source(s)^c^**	Pharmacist	33.2% (189)	38.2% (143)	23.6% (46)	-14.6% [-22.2, -6.7]	<0.001*
	Other healthcare provider	8.4% (48)	9.6% (36)	6.2% (12)	-3.5% [-7.8, 1.4]	0.209
	Another source	7.6% (43)	8.6% (32)	5.6% (11)	-2.9% [-7.1, 1.7]	0.279
	I have never received this information	42.9% (244)	34.8% (130)	58.5% (114)	23.7% [15.1, 31.9]	<0.001*
	I don’t recall	11.4% (65)	12.3% (46)	9.7% (19)	-2.6% [-7.7, 3.0]	0.441
**Received disposal information from a pharmacist or other healthcare provider**	—	37.4% (213)	42.8% (160)	27.2% (53)	-15.6% [-23.4, -7.4]	<0.001*
**Opioid handling behaviors**						
**Do you have any opioid medication in your household that is no longer being used or is expired?^c^**	Yes, medication prescribed to me	30.6% (174)	—	89.2% (174)	—	—
	Yes, medication prescribed to someone else	5.8% (33)	—	16.9% (33)	—	
**How do you store opioid/narcotic medication in your household?^a^**	Locked storage	9.8% (52)	13.4% (45)	3.7% (7)	-9.7% [-14.0, -4.8]	<0.001*
	Unlocked storage	90.2% (476)	86.6% (292)	96.3% (184)	9.7% [4.8, 14.0]	
**How do you dispose of your unused opioid medication?^c^**	Flush down the sink or toilet	7.9% (45)	8.8% (33)	6.2% (12)	-2.7% [-7.0, 2.1]	0.339
	Throw away in garbage	15.5% (88)	11.8% (44)	22.6% (44)	10.8% [4.2, 17.6]	0.001*
	Mix with undesirable material (e.g. kitty litter, coffee grounds) and throw in garbage	1.8% (10)	2.1% (8)	1.0% (2)	-1.1% [-3.2, 1.4]	0.533
	Return it to the pharmacy or a community take-back program	54.1% (308)	56.7% (212)	49.2% (96)	-7.5% [-16.0, 1.2]	0.109
	I would not dispose of it	13.5% (77)	9.9% (37)	20.5% (40)	10.6% [4.3, 17.1]	<0.001*
	Other method	5.4% (31)	4.8% (18)	6.7% (13)	1.9% [-2.2, 6.3]	0.465
**Respondent reported a safe disposal method**	—	55.5% (316)	58.3% (218)	50.3% (98)	-8.0% [-16.6, 0.6]	0.082
**Have you ever shared prescription opioid/narcotic medication with another person?^b^**	Yes, I have shared medication prescribed to me	4.6% (26)	3.2% (12)	7.2% (14)	4.0% [-0.9, 9.2]	<0.001*
	Yes, I have used medication prescribed to someone else	2.1% (12)	1.1% (4)	4.1% (8)	3.0% [-0.6, 7.1]	
	Yes, I have both shared medication prescribed to me and used medication prescribed to someone else	1.1% (6)	0.3% (1)	2.6% (5)	2.3% [-0.6, 5.6]	
	No	92.3% (525)	95.5% (357)	86.2% (168)	-9.3% [-15.9, -2.9]	
**If you had unused opioid/narcotic medication, would you be willing to bring it to your next appointment at the hospital for disposal?^a^**	Yes (versus no)	71.7% (377)	75.1% (251)	65.6% (126)	-9.5% [-17.7, -1.4]	0.026*

CI = confidence interval.

* P < 0.05 (chi-square test of independence: No unused opioids versus any unused opioids).

^a^ Percentages calculated excluding missing responses.

^b^ Confidence intervals adjusted using Bonferroni correction to control familywise error.

^c^ Multiple selections were permitted; percentages may total more than 100%.

Many respondents reported unsafe opioid handling practices. Only 10% of respondents reported using a secure (locked) storage location; 90% reported an unlocked storage location. While 56% said they typically used a Health Canada-recommended disposal method [12] (i.e., return to the pharmacy or a take-back program), 44% reported neither of the recommended methods, with 14% reporting that they would not dispose of unused opioid medication. Eight percent reported sharing opioid medication (i.e., letting others use opioids prescribed to them and/or using opioids prescribed to someone else). Compared to respondents with no unused opioids, those with unused opioids at home were more likely to report unsafe handling practices, including storing opioids in an unsecured location (96% vs. 87% using unlocked storage, p = 0.001); disposing of opioids by throwing them in the garbage (23% vs. 12%, p = 0.001); stating that they would not dispose of opioids at all (21% vs 10%, p<0.001); and sharing prescription opioids (14% vs. 5%, p<0.001). Respondents in our sample who received storage and disposal instructions from a pharmacist or health care provider did report safer handling behaviors: they were less likely to have unused opioids at home (p = 0.005), more likely to keep opioids in a locked location (p<0.001), and more likely to dispose of opioids at a pharmacy or take-back program (p<0.001) (see S1A–S1C Table for comparative analysis of those who reported receiving instruction versus those who did not).

Seventy-two percent of respondents indicated they would be willing to return unused opioids in a potential hospital-based take-back program. Respondents with unused opioids were less willing to participate (66% vs. 75%, p = 0.026) (Table 2). Among the 28% of respondents who would not participate in the hypothetical program, the most common reasons given were preferring to dispose of the medication on their own (40%), concern about obtaining opioids again in a timely manner (29%), and fear that their pain would return (24%) (Table 3). Compared to respondents with no unused opioids, those with unused opioids at home were more likely to report fear that their pain would return (33% vs. 16%, p = 0.020); other factors were not significantly different between the two groups.

**Table 3:** Reasons for not wanting to participate in an opioid take-back program, overall and by presence of unused opioids at home.

		Unused opioid medication at home			
Response^a^	All respondents unwilling to participate, % (count) N = 149	No unused opioids, % (count) N = 83	Any unused opioids, % (count) N = 66	Difference [95% CI]	P
**I am afraid my pain will come back and I’m not willing to be without it**	23.5% (35)	15.7% (13)	33.3% (22)	17.7% [3.6, 31.1]	0.020*
**If I need it again, I will not be able to get it from a doctor in a timely manner**	28.9% (43)	26.5% (22)	31.8% (21)	5.3% [-9.3, 19.9]	0.597
**I do not want to throw it away because I paid for it**	10.1% (15)	6.0% (5)	15.2% (10)	9.1% [-1.2, 19.4]	0.118
**My friends or family members may need it**	3.4% (5)	3.6% (3)	3.0% (2)	-0.6% [-6.9, 6.3]	1.000
**I would prefer to dispose of it myself**	38.9% (58)	39.8% (33)	37.9% (25)	-1.9% [-17.3, 13.8]	0.948
**Other reason**	17.4% (26)	21.7% (18)	12.1% (8)	-9.6% [-21.1, 2.9]	0.190

CI = confidence interval.

* P < 0.05 (chi-square test of independence: No unused opioids versus any unused opioids).

^a^ Multiple selections were permitted; percentages may total more than 100%.

The presence of minors or young adults in the household was not associated with using a secure opioid storage location (Table 4). Compared to respondents who used a locking opioid storage method, respondents who used non-locking storage methods reported similar rates of minors and young adults in the home. Of the 476 respondents who reported storing opioids in an unlocked location, 21% had at least one child (age 0 to 12) and 29% had at least one teenager or young adult (age 13 to 25) in the home.

**Table 4:** Presence of children, teenagers and/or young adults in the household versus opioid storage method, among current and former opioid users.

		Opioid storage type ^a^		
	Age group	Locked (N = 52)	Not locked (N = 476)	P
**Do children, teenagers, or young adults live in your home or visit your home? ^b^**				
	No children/teenagers/young adults reported	57.7% (30)	56.5% (269)	0.988
	Young children (age 0–6)	11.5% (6)	13.0% (62)	0.932
	Older children (age 7–12)	17.3% (9)	14.1% (67)	0.673
	Teenagers (age 13–17)	19.2% (10)	12.0% (57)	0.203
	Young adults (age 18–25)	19.2% (10)	21.8% (104)	0.796
**Combined age groups:**				
	Derived: Any children (age 0–12)	23.1% (12)	20.6% (98)	0.811
	Derived: Any teenagers or young adults (age 13–25)	32.7% (17)	28.8% (137)	0.668

^a^ 41 respondents who did not report their storage method were excluded.

^b^ Multiple selections were permitted; percentages may total more than 100%.

* P < 0.05 (chi-square test of independence: Locked storage versus non-locked storage).

## Discussion

This study reveals that many opioid-using orthopaedic patients have unsafe opioid handling practices, and report not receiving instruction on safely managing their prescription opioids from their pharmacist or HCP. In particular, patients with unused opioids in the home reported lower instruction rates, and almost half of this group had minors or young adults in the household. Most orthopaedic patients are willing to participate in a hospital-based opioid disposal program.

We found that orthopaedic patients frequently stored and disposed of opioids incorrectly, consistent with findings from U.S. studies [20]. Only 10% of respondents stored opioids securely, and only 41% of respondents disposed of unused opioids appropriately. A recent systematic review with 810 surgical patients concluded that 77% of patients insecurely stored their opioids and only 9% of patients followed the FDA-recommended disposal methods [21]. Improper storage and retention of unused opioids both increase the risk of opioid misuse, diversion, and accidental poisoning [22,23]. Suboptimal disposal may also contaminate environmental reservoirs [24,25]. Medication take-back programs and coordinated initiatives, such as National Prescription Drug Drop-Off Day in Canada, have been established to promote safe storage and disposal practices and to reduce the number of unused tablets readily available within the community [26]. While promising, these programs tend to have poor uptake and remain in their rudimentary stages of implementation [27,28]. Furthermore, to the best of our knowledge, these programs are seldom promoted in the hospital setting.

While the orthopaedic patients in our study did not explicitly report the reason for their opioid use, it is likely that many of their prescriptions were related to orthopaedic conditions. Short-term opioid use remains essential in the management of postoperative pain or acute orthopaedic injuries [29–34]. Therefore, in order to minimize opioid diversion opportunities, it is imperative that patients receive adequate education regarding safe storage and disposal of opioids. Only a third of patients in our sample recalled receiving instructions on safe opioid handling practices from their pharmacist, and only 10% from other healthcare providers. Other studies in both surgery and emergency departments have found similar low rates of instruction [35–38]. These results suggest a need for both hospital- and community-based HCPs to address not only prescribing practices [10,11,39], but also patients’ opioid handling knowledge (and ultimately, behaviour).

Consistent with other studies, one third of surveyed patients reported having opioids at home that were no longer being used [20,40–42]. We found that these patients were less willing to participate in a hospital-based take-back program compared to those with no unused opioids, citing fear that their pain would return, concerns about untimely access to more opioids, and that they did not want to dispose of medication they paid for. Furthermore, these patients were less likely to report receiving opioid handling education and using safe handling methods, and almost half of the group reported having minors or young adults in the household. This combination of factors may signify a high risk of opioid diversion in this group.

In our study, 8% of current or former opioid users reported sharing their medications with others, and of the 90% who stored their opioids in an unsecured location, 44% reported children, teenagers, or young adults in their household. Previous research indicates that drug misuse is directly linked to the presence of leftover medications in unsecured household cabinets and through sharing unused medications [22,37,43–45]. In fact, 70%-75% of abusers obtained opioids through methods of diversion and only 5% from drug dealers or strangers [46–51]. Consistent with our findings, recent studies have demonstrated that prior instruction in safe opioid handling practices was highly associated with returning medications to a pharmacy, and was the factor most strongly associated with returning medications to a clinician [52–57]. Consequently, it is essential that orthopaedic surgeons, as well as other hospital and community HCPs allocate sufficient time toward educating patients on safe opioid handling practices and the potential danger of opioid diversion. Our subsequent work will aim to determine whether a hospital-based education program is effective for promoting safer opioid handling among patients.

## Limitations

This study had several limitations. Media attention and stigma surrounding opioids may have contributed to a social desirability bias, leading participants to underreport opioid use, retention of unused opioids, and unsafe handling practices. An anonymous survey and survey return mechanism were used to minimize this effect. Recall bias may also lead participants to misreport prior education on opioid handling. Second, the voluntary nature of the questionnaire creates a risk of selection bias. The representativeness of the sample cannot be confirmed, as the number of patients who declined to participate was not tracked. Although the sample’s age and gender distribution is similar to that of the general orthopaedic population [58], the study was conducted in a single large, urban, tertiary care centre which may limit the generalizability of the findings. Lastly, the study data was collected at a single time point; patients’ opioid handling practices may have changed with increased awareness of the opioid epidemic.

## Conclusion

Patients attending an ambulatory Orthopedic clinic who have been prescribed opioids commonly report possession of unused opioids and unsafe opioid handling practices. As well, relatively few patients prescribed opioids report receiving education on safe opioid handling practices from their pharmacist, and even fewer from other healthcare providers. Our findings reveal a significant opportunity for hospital-driven opioid stewardship. While overprescribing of opioids remains a critical issue, our study suggests a concurrent need to address patient knowledge and behavior regarding opioid handling in order to reduce the risk of opioid diversion in the households of opioid-using patients.

## Supporting information

S1 Table Comparative analysis of opioid-using respondents by receipt of opioid storage and disposal instruction. (PDF)

S1 Appendix Public safety survey. (PDF)

## References

[1] Belzak L, Halverson J. The opioid crisis in Canada: a national perspective. Health Promot Chronic Dis Prev Can Res Policy Pract. 2018;38: 224–233. doi: 10.24095/hpcdp.38.6.02 29911818 PMC6034966

[2] Special Advisory Committee on the Epidemic of Opioid Overdoses. Opioids and stimulant-related Harms in Canada. Ottawa: Public Health Agency of Canada; 2020. Available: https://health-infobase.canada.ca/substance-related-harms/opioids-stimulants.

[3] Canadian Institute for Health Information. Opioid-Related Harms in Canada. Ottawa, ON: CIHI; 2018. Available: https://secure.cihi.ca/free_products/opioid-related-harms-report-2018-en-web.pdf.

[4] Boak A, Elton-Marshall T, Mann RE, Hamilton HA. Drug use among Ontario students, 1977–2019: Detailed findings from the Ontario Student Drug Use and Health Survey (OSDUHS. Toronto, ON: Centre for Addiction and Mental Health; 2020. Available: https://www.camh.ca//-/media/files/pdf---osduhs/drugusereport_2019osduhs-pdf.pdf?la=en%26hash=7F149240451E7421C3991121AEAD630F21B13784.

[5] Bedson J, Chen Y, Hayward RA, Ashworth J, Walters K, Dunn KM, et al. Trends in long-term opioid prescribing in primary care patients with musculoskeletal conditions: an observational database study. Pain. 2016;157: 1525–1531. doi: 10.1097/j.pain.0000000000000557 27003191 PMC4912234

[6] Ladha KS, Neuman MD, Broms G, Bethell J, Bateman BT, Wijeysundera DN, et al. Opioid Prescribing After Surgery in the United States, Canada, and Sweden. JAMA Netw Open. 2019;2: e1910734. doi: 10.1001/jamanetworkopen.2019.10734 31483475 PMC6727684

[7] Ashaye T, Hounsome N, Carnes D, Taylor SJC, Homer K, Eldridge S, et al. Opioid prescribing for chronic musculoskeletal pain in UK primary care: results from a cohort analysis of the COPERS trial. BMJ Open. 2018;8: e019491. doi: 10.1136/bmjopen-2017-019491 29880563 PMC6009475

[8] Morris BJ, Mir HR. The opioid epidemic: impact on orthopaedic surgery. J Am Acad Orthop Surg. 2015;23: 267–271. doi: 10.5435/JAAOS-D-14-00163 25911660

[9] Thiels CA, Anderson SS, Ubl DS, Hanson KT, Bergquist WJ, Gray RJ, et al. Wide Variation and Overprescription of Opioids After Elective Surgery. Ann Surg. 2017. doi: 10.1097/SLA.0000000000002365 28697049

[10] Health Quality Ontario. Opioid Prescribing for Chronic Pain: Care for People 15 Years of Age and Older. Toronto, ON: Health Quality Ontario; 2018 p. 52. Available: https://www.hqontario.ca/portals/0/documents/evidence/quality-standards/qs-opioid-chronic-pain-clinician-guide-en.pdf.

[11] Canadian Institute for Health Information. Opioid Prescribing in Canada: How Are Practices Changing? Ottawa, ON: CIHI; 2019 p. 42. Available: https://www.cihi.ca/sites/default/files/document/opioid-prescribing-canada-trends-en-web.pdf.

[12] Government of Canada. About Opioids. Ottawa, ON; 2019. Available: https://www.canada.ca/en/health-canada/services/substance-use/problematic-prescription-drug-use/opioids/about.html.

[13] Government of Canada. Safe disposal of prescription drugs. 2017 [cited 24 May 2017]. Available: https://www.canada.ca/en/health-canada/services/safe-disposal-prescription-drugs.html.

[14] Tanabe P, Paice JA, Stancati J, Fleming M. How do emergency department patients store and dispose of opioids after discharge? A pilot study. J Emerg Nurs JEN Off Publ Emerg Dep Nurses Assoc. 2012;38: 273–279. doi: 10.1016/j.jen.2011.09.023 22204885

[15] Reddy A, de la Cruz M, Rodriguez EM, Thames J, Wu J, Chisholm G, et al. Patterns of storage, use, and disposal of opioids among cancer outpatients. The Oncologist. 2014;19: 780–785. doi: 10.1634/theoncologist.2014-0071 24868100 PMC4077453

[16] Kennedy-Hendricks A, Gielen A, McDonald E, McGinty EE, Shields W, Barry CL. Medication Sharing, Storage, and Disposal Practices for Opioid Medications Among US Adults. JAMA Intern Med. 2016;176: 1027–1029. doi: 10.1001/jamainternmed.2016.2543 27295629

[17] McDonald EM, Kennedy-Hendricks A, McGinty EE, Shields WC, Barry CL, Gielen AC. Safe Storage of Opioid Pain Relievers Among Adults Living in Households With Children. Pediatrics. 2017;139: e20162161. doi: 10.1542/peds.2016-2161 28219969

[18] Agresti A, Caffo B. Simple and Effective Confidence Intervals for Proportions and Differences of Proportions Result from Adding Two Successes and Two Failures. Am Stat. 2000;54: 280–288. doi: 10.1080/00031305.2000.10474560

[19] Dunn OJ. Multiple Comparisons among Means. J Am Stat Assoc. 1961;56: 52–64. doi: 10.1080/01621459.1961.10482090

[20] Sabatino MJ, Kunkel ST, Ramkumar DB, Keeney BJ, Jevsevar DS. Excess Opioid Medication and Variation in Prescribing Patterns Following Common Orthopaedic Procedures. J Bone Joint Surg Am. 2018;100: 180–188. doi: 10.2106/JBJS.17.00672 29406338 PMC6818977

[21] Bicket MC, Long JJ, Pronovost PJ, Alexander GC, Wu CL. Prescription Opioid Analgesics Commonly Unused After Surgery: A Systematic Review. JAMA Surg. 2017;152: 1066–1071. doi: 10.1001/jamasurg.2017.0831 28768328 PMC5701659

[22] Kinrys G, Gold AK, Worthington JJ, Nierenberg AA. Medication disposal practices: Increasing patient and clinician education on safe methods. J Int Med Res. 2018;46: 927–939. doi: 10.1177/0300060517738681 29322845 PMC5972255

[23] McCabe SE, West BT, Veliz P, McCabe VV, Stoddard SA, Boyd CJ. Trends in Medical and Nonmedical Use of Prescription Opioids Among US Adolescents: 1976–2015. Pediatrics. 2017;139: e20162387. doi: 10.1542/peds.2016-2387 28320868 PMC5369669

[24] Daughton CG, Ruhoy IS. Environmental footprint of pharmaceuticals: the significance of factors beyond direct excretion to sewers. Environ Toxicol Chem. 2009;28: 2495–2521. doi: 10.1897/08-382.1 19382823

[25] Glassmeyer ST, Hinchey EK, Boehme SE, Daughton CG, Ruhoy IS, Conerly O, et al. Disposal practices for unwanted residential medications in the United States. Environ Int. 2009;35: 566–572. doi: 10.1016/j.envint.2008.10.007 19081631

[26] Wu PE, Juurlink DN. Unused prescription drugs should not be treated like leftovers. CMAJ Can Med Assoc J J Assoc Medicale Can. 2014;186: 815–816. doi: 10.1503/cmaj.140222 24799553 PMC4119135

[27] Kozak MA, Melton JR, Gernant SA, Snyder ME. A needs assessment of unused and expired medication disposal practices: A study from the Medication Safety Research Network of Indiana. Res Soc Adm Pharm RSAP. 2016;12: 336–340. doi: 10.1016/j.sapharm.2015.05.013 26143488 PMC5221553

[28] Egan KL, Gregory E, Sparks M, Wolfson M. From dispensed to disposed: evaluating the effectiveness of disposal programs through a comparison with prescription drug monitoring program data. Am J Drug Alcohol Abuse. 2017;43: 69–77. doi: 10.1080/00952990.2016.1240801 27797283

[29] Wunsch H, Wijeysundera DN, Passarella MA, Neuman MD. Opioids Prescribed After Low-Risk Surgical Procedures in the United States, 2004–2012. JAMA. 2016;315: 1654–1657. doi: 10.1001/jama.2016.0130 26978756 PMC4837043

[30] Fandino LB, Bhashyam A, Harris MB, Zhang D. Factors associated with discharge opioid prescription after hip fracture fixation. Musculoskeletal Care. 2020;18: 352–358. doi: 10.1002/msc.1466 32202702

[31] Stratton A, Wai E, Kingwell S, Phan P, Roffey D, El Koussy M, et al. Opioid use trends in patients undergoing elective thoracic and lumbar spine surgery. Can J Surg J Can Chir. 2020;63: E306–E312. doi: 10.1503/cjs.018218 32463627 PMC7829003

[32] Merrill HM, Dean DM, Mottla JL, Neufeld SK, Cuttica DJ, Buchanan MM. Opioid Consumption Following Foot and Ankle Surgery. Foot Ankle Int. 2018;39: 649–656. doi: 10.1177/1071100718757527 29506395

[33] Warner NS, Habermann EB, Hooten WM, Hanson AC, Schroeder DR, St Sauver JL, et al. Association Between Spine Surgery and Availability of Opioid Medication. JAMA Netw Open. 2020;3: e208974. doi: 10.1001/jamanetworkopen.2020.8974 32584410 PMC7317600

[34] Hsu JR, Mir H, Wally MK, Seymour RB, Orthopaedic Trauma Association Musculoskeletal Pain Task Force. Clinical Practice Guidelines for Pain Management in Acute Musculoskeletal Injury. J Orthop Trauma. 2019;33: e158–e182. doi: 10.1097/BOT.0000000000001430 30681429 PMC6485308

[35] Kumar K, Gulotta LV, Dines JS, Allen AA, Cheng J, Fields KG, et al. Unused Opioid Pills After Outpatient Shoulder Surgeries Given Current Perioperative Prescribing Habits. Am J Sports Med. 2017;45: 636–641. doi: 10.1177/0363546517693665 28182507

[36] Garren BR, Lawrence MB, McNaull PP, Sutherland R, Bukowski TP, Nielsen ME, et al. Opioid-prescribing patterns, storage, handling, and disposal in postoperative pediatric urology patients. J Pediatr Urol. 2019;15: 260.e1-260.e7. doi: 10.1016/j.jpurol.2019.02.009 31010641

[37] Silvestre J, Reddy A, de la Cruz M, Wu J, Liu D, Bruera E, et al. Frequency of unsafe storage, use, and disposal practices of opioids among cancer patients presenting to the emergency department. Palliat Support Care. 2017;15: 638–643. doi: 10.1017/S1478951516000158 27071690

[38] Buffington DE, Lozicki A, Alfieri T, Bond TC. Understanding factors that contribute to the disposal of unused opioid medication. J Pain Res. 2019;12: 725–732. doi: 10.2147/JPR.S171742 30863145 PMC6388750

[39] Shah A, Hayes CJ, Martin BC. Factors Influencing Long-Term Opioid Use Among Opioid Naive Patients: An Examination of Initial Prescription Characteristics and Pain Etiologies. J Pain. 2017;18: 1374–1383. doi: 10.1016/j.jpain.2017.06.010 28711636 PMC5660650

[40] Dautremont EA, Ebramzadeh E, Beck JJ, Bowen RE, Sangiorgio SN. Opioid Prescription and Usage in Adolescents Undergoing Orthopaedic Surgery in the United States: A Systematic Review. JBJS Rev. 2017;5: e5. doi: 10.2106/JBJS.RVW.16.00093 28796696

[41] Bhashyam AR, Young J, Qudsi RA, Parisien RL, Dyer GSM. Opioid Prescribing Patterns of Orthopedic Surgery Residents After Open Reduction Internal Fixation of Distal Radius Fractures. J Hand Surg. 2019;44: 201–207.e2. doi: 10.1016/j.jhsa.2018.11.003 30635200

[42] Kim N, Matzon JL, Abboudi J, Jones C, Kirkpatrick W, Leinberry CF, et al. A Prospective Evaluation of Opioid Utilization After Upper-Extremity Surgical Procedures: Identifying Consumption Patterns and Determining Prescribing Guidelines. J Bone Joint Surg Am. 2016;98: e89. doi: 10.2106/JBJS.15.00614 27869630

[43] Kinrys G, K Gold A, Nierenberg AA. Proper Drug Disposal: Studying a Solution to Household Prescription and Over-the- Counter Drug Abuse. J Drug Abuse. 2016;02. doi: 10.21767/2471-853X.100027

[44] Yang C, Stilley JAW, Bedy S-MC, Goddard KB, Sampson CS. Leftover narcotic analgesics among emergency department patients and methods of disposal. J Am Coll Emerg Physicians Open. 2020;1: 1486–1492. doi: 10.1002/emp2.12161 33392554 PMC7771740

[45] Bicket MC, White E, Pronovost PJ, Wu CL, Yaster M, Alexander GC. Opioid Oversupply After Joint and Spine Surgery: A Prospective Cohort Study. Anesth Analg. 2019;128: 358–364. doi: 10.1213/ANE.0000000000003364 29677062

[46] Manchikanti L, Helm S, Fellows B, Janata JW, Pampati V, Grider JS, et al. Opioid epidemic in the United States. Pain Physician. 2012;15: ES9–38. 22786464

[47] Maxwell JC. The prescription drug epidemic in the United States: a perfect storm. Drug Alcohol Rev. 2011;30: 264–270. doi: 10.1111/j.1465-3362.2011.00291.x 21545556

[48] Han B, Compton WM, Blanco C, Crane E, Lee J, Jones CM. Prescription Opioid Use, Misuse, and Use Disorders in U.S. Adults: 2015 National Survey on Drug Use and Health. Ann Intern Med. 2017;167: 293–301. doi: 10.7326/M17-0865 28761945

[49] Lewis ET, Cucciare MA, Trafton JA. What do patients do with unused opioid medications? Clin J Pain. 2014;30: 654–662. doi: 10.1097/01.ajp.0000435447.96642.f4 24281287

[50] Kaye AD, Jones MR, Kaye AM, Ripoll JG, Jones DE, Galan V, et al. Prescription Opioid Abuse in Chronic Pain: An Updated Review of Opioid Abuse Predictors and Strategies to Curb Opioid Abuse (Part 2). Pain Physician. 2017;20: S111–S133. 28226334

[51] Volkow N, Benveniste H, McLellan AT. Use and Misuse of Opioids in Chronic Pain. Annu Rev Med. 2018;69: 451–465. doi: 10.1146/annurev-med-011817-044739 29029586

[52] de la Cruz M, Reddy A, Balankari V, Epner M, Frisbee-Hume S, Wu J, et al. The Impact of an Educational Program on Patient Practices for Safe Use, Storage, and Disposal of Opioids at a Comprehensive Cancer Center. The Oncologist. 2017;22: 115–121. doi: 10.1634/theoncologist.2016-0266 27742907 PMC5313268

[53] Bettlach CLR, Hasak JM, Santosa KB, Larson EL, Tung TH, Fox IK, et al. A Simple Brochure Improves Disposal of Unused Opioids: An Observational Cross-Sectional Study. Hand N Y N. 2020; 1558944720959898. doi: 10.1177/1558944720959898 33025827 PMC8721787

[54] Rose P, Sakai J, Argue R, Froehlich K, Tang R. Opioid information pamphlet increases postoperative opioid disposal rates: a before versus after quality improvement study. Can J Anaesth J Can Anesth. 2016;63: 31–37. doi: 10.1007/s12630-015-0502-0 26431852

[55] Hasak JM, Roth Bettlach CL, Santosa KB, Larson EL, Stroud J, Mackinnon SE. Empowering Post-Surgical Patients to Improve Opioid Disposal: A Before and After Quality Improvement Study. J Am Coll Surg. 2018;226: 235–240.e3. doi: 10.1016/j.jamcollsurg.2017.11.023 29331347 PMC5826881

[56] Varisco TJ, Fleming ML, Bapat SS, Wanat MA, Thornton D. Health care practitioner counseling encourages disposal of unused opioid medications. J Am Pharm Assoc JAPhA. 2019;59: 809–815.e5. doi: 10.1016/j.japh.2019.07.010 31474526

[57] Nahhas CR, Hannon CP, Yang J, Gerlinger TL, Nam D, Della Valle CJ. Education Increases Disposal of Unused Opioids After Total Joint Arthroplasty: A Cluster-Randomized Controlled Trial. J Bone Joint Surg Am. 2020;102: 953–960. doi: 10.2106/JBJS.19.01166 32251139

[58] Canizares M, Davis AM, Badley EM. The pathway to orthopaedic surgery: a population study of the role of access to primary care and availability of orthopaedic services in Ontario, Canada. BMJ Open. 2014;4: e004472. doi: 10.1136/bmjopen-2013-004472 25082417 PMC4120425

