# Supplementary material for: The potential for diversion of prescribed opioids among orthopaedic patients: Results of an anonymous patient survey
Source: PLoS One. 2021 Aug 26;16(8):e0256741. doi: 10.1371/journal.pone.0256741 (PMC8389484; doi:10.1371/journal.pone.0256741)
Supplement: S1 Table — (PDF) [file pone.0256741.s001.pdf]

S1 Table. Comparative analysis of opioid-using respondents by receipt of opioid storage and disposal instruction.

Table S1A. Characteristics of opioid-using respondents, overall and by respondent's opioid education status.

			Received information from pharmacist or HCP on safe storage and disposal of opioids, % (count)			
**Measure**	**Category**	**All opioid ever-users, % (count) N = 569**	**Did not receive information N = 407**	**Received information N = 162**	**Difference [95% CI]**	**P**
**Age group[a,b]**	18-24	3.9% (22)	4.4% (18)	2.5% (4)	-2.0% [-6.1, 2.9]	0.643
	25-34	11.1% (63)	12.3% (50)	8.0% (13)	-4.3% [-11.2, 3.2]	
	35-44	10.7% (61)	11.1% (45)	9.9% (16)	-1.2% [-8.4, 6.6]	
	45-54	16.7% (95)	16.0% (65)	18.5% (30)	2.5% [-6.7, 12.1]	
	55-64	27.8% (158)	27.1% (110)	29.6% (48)	2.5% [-8.4, 13.7]	
	65-74	21.1% (120)	20.4% (83)	22.8% (37)	2.4% [-7.6, 12.8]	
	75+	8.6% (49)	8.6% (35)	8.6% (14)	0.0% [-6.7, 7.3]	
**Female sex[a]**	—	59.6% (334)	59.4% (238)	60.4% (96)	1.0% [-8.0, 9.9]	0.898
**Prescription opioid medication use[b]**	Former	71.5% (407)	75.7% (308)	61.1% (99)	-14.6% [-24.4, -4.8]	<0.001*
	Sometimes	11.8% (67)	11.1% (45)	13.6% (22)	2.5% [-4.2, 9.8]	
	Daily	16.7% (95)	13.3% (54)	25.3% (41)	12.0% [3.6, 20.7]	
**Do children, teenagers, or young adults live in your home or visit your home?[c]**	Young children (age 0-6)	12.3% (70)	11.3% (46)	14.8% (24)	3.5% [-2.6, 10.1]	0.313
	Older children (age 7-12)	13.5% (77)	12.5% (51)	16.0% (26)	3.5% [-2.8, 10.3]	0.331
	Teenagers (age 13-17)	12.0% (68)	12.0% (49)	11.7% (19)	-0.3% [-6.0, 5.9]	1.000
	Young adults (age 18-25)	20.7% (118)	21.6% (88)	18.5% (30)	-3.1% [-10.1, 4.3]	0.478
	None of the above	58.3% (332)	58.0% (236)	59.3% (96)	1.3% [-7.7, 10.1]	0.854

HCP = health care provider; CI = confidence interval

* P < 0.05 (chi-square test of independence: did not receive information versus received information)

[a] Percentages calculated excluding missing responses.

[b] Confidence intervals adjusted using Bonferroni correction to control familywise error.

[c] Multiple selections were permitted; percentages may total more than 100%.

Table S1B. Opioid handling behaviours, overall and by respondent's opioid education status.

			Received information from pharmacist or HCP on safe storage and disposal of opioids, % (count)			
**Measure**	**Category**	**All opioid ever-users, % (count) N = 569**	**Did not receive information N = 407**	**Received information N = 162**	**Difference [95% CI]**	**P**
**Do you have any opioid medication in your household that is no longer being used or is expired?[c]**	Yes, medication prescribed to me	30.6% (174)	34.2% (139)	21.6% (35)	-12.5% [-20.1, -4.5]	0.005*
	Yes, medication prescribed to someone else	5.8% (33)	7.1% (29)	2.5% (4)	-4.7% [-7.9, -0.6]	0.052
**How do you store opioid/narcotic medication in your household?[a]**	Locked storage	9.8% (52)	4.8% (18)	22.4% (34)	17.6% [10.7, 24.7]	<0.001*
	Unlocked storage	90.2% (476)	95.2% (358)	77.6% (118)	-17.6% [-24.7, -10.7]	
**How do you dispose of your unused opioid medication?[c]**	Flush down the sink or toilet	7.9% (45)	8.4% (34)	6.8% (11)	-1.6% [-6.1, 3.6]	0.652
	Throw away in garbage	15.5% (88)	19.7% (80)	4.9% (8)	-14.7% [-19.5, -9.1]	<0.001*
	Mix with undesirable material (e.g. kitty litter, coffee grounds) and throw in garbage	1.8% (10)	2.2% (9)	0.6% (1)	-1.6% [-3.5, 1.0]	0.341
	Return it to the pharmacy or a community take-back program	54.1% (308)	44.0% (179)	79.6% (129)	35.6% [27.4, 43.1]	<0.001*
	I would not dispose of it	13.5% (77)	17.4% (71)	3.7% (6)	-13.7% [-18.2, -8.5]	
	Other method	5.4% (31)	6.4% (26)	3.1% (5)	-3.3% [-6.7, 0.8]	0.173

			Received information from pharmacist or HCP on safe storage and disposal of opioids, % (count)			
**Measure**	**Category**	**All opioid ever-users, % (count) N = 569**	**Did not receive information N = 407**	**Received information N = 162**	**Difference [95% CI]**	**P**
**Have you ever shared prescription opioid/narcotic medication with another person?[b]**	Yes, I have shared medication prescribed to me	4.6% (26)	5.2% (21)	3.1% (5)	-2.1% [-6.1, 2.7]	0.292
	Yes, I have used medication prescribed to someone else	2.1% (12)	2.0% (8)	2.5% (4)	0.5% [-2.8, 4.5]	
	Yes, I have both shared medication prescribed to me and used medication prescribed to someone else	1.1% (6)	1.5% (6)	0.0% (0)	-1.5% [-3.2, 1.0]	
	No	92.3% (525)	91.4% (372)	94.4% (153)	3.0% [-2.9, 8.3]	
**If you had unused opioid/narcotic medication, would you be willing to bring it to your next appointment at the hospital for disposal?[a]**	Yes (versus no)	71.7% (377)	72.8% (275)	68.9% (102)	-3.8% [-12.6, 4.7]	0.442

HCP = health care provider; CI = confidence interval

* P < 0.05 (chi-square test of independence: did not receive information versus received information)

[a] Percentages calculated excluding missing responses.

[b] Confidence intervals adjusted using Bonferroni correction to control familywise error.

[c] Multiple selections were permitted; percentages may total more than 100%.

Table S1C. Reasons for not wanting to participate in an opioid take-back program, overall and by respondent's opioid education status.

		Received information from pharmacist or HCP on safe storage and disposal of opioids, % (count)			
**Response[a]**	**All respondents unwilling to participate, % (count) N = 149**	**Did not receive information N = 103**	**Received information N = 46**	**Difference [95% CI]**	**P**
**I am afraid my pain will come back and I'm not willing to be without it**	23.5% (35)	27.2% (28)	15.2% (7)	-12.0% [-24.5, 2.6]	0.167
**If I need it again, I will not be able to get it from a doctor in a timely manner**	28.9% (43)	29.1% (30)	28.3% (13)	-0.9% [-15.9, 15.2]	1.000
**I do not want to throw it away because I paid for it**	10.1% (15)	12.6% (13)	4.3% (2)	-8.3% [-16.5, 2.4]	0.209
**My friends or family members may need it**	3.4% (5)	3.9% (4)	2.2% (1)	-1.7% [-7.6, 6.4]	0.966
**I would prefer to dispose of it myself**	38.9% (58)	36.9% (38)	43.5% (20)	6.6% [-10.2, 23.4]	0.562
**Other reason**	17.4% (26)	14.6% (15)	23.9% (11)	9.3% [-4.3, 23.8]	0.248

HCP = health care provider; CI = confidence interval

* $P < 0.05$ (chi-square test of independence: did not receive information versus received information)

[a] Multiple selections were permitted; percentages may total more than 100%.
