# Supplementary material for: The potential for diversion of prescribed opioids among orthopaedic patients: Results of an anonymous patient survey
Source: PLoS One. 2021 Aug 26;16(8):e0256741. doi: 10.1371/journal.pone.0256741 (PMC8389484; doi:10.1371/journal.pone.0256741)
Supplement: S1 Appendix — (PDF) [file pone.0256741.s002.pdf]

S1 Appendix: Public Safety Survey

As part of the development of a public safety initiative, we would like to improve our understanding of how patients use, store, and dispose of prescription opioid pain medications (also known as narcotic pain medications). This survey is **anonymous** and all individual responses will be kept strictly confidential. Mark your responses using an ‘x’.

The table below shows some common types and names of opioid/narcotic medications:

Medication	Common brand names
**Codeine**	Tylenol #2, Tylenol #3, Tylenol #4
**Fentanyl**	Actiq, Duragesic, Fentora
**Hydrocodone**	Hysingla ER, Zohydro ER, Lorcet, Lortab, Norco, Vicodin
**Hydromorphone**	Dilaudid, Exalgo
**Meperidine**	Demerol
**Methadone**	Dolophine, Methadose
**Morphine**	Astramorph, Avinza, Kadian, MS Contin, Ora-Morph SR
**Oxycodone**	OxyContin, Oxecta, Roxicodone, Percocet, Endocet, Roxicet, Targiniq ER

1. What is your age group? Select one:

◯ 18 to 24 ◯ 25 to 34 ◯ 35 to 44 ◯ 45 to 54 ◯ 55 to 64 ◯ 65 to 74 ◯ 75+

2. What is your sex? Select one:

◯ Female ◯ Male

3. Have you ever been prescribed any opioid/narcotic pain medication? Select one:

◯ I have never been prescribed opioid/narcotic pain medication ⟶ ***go to question 4***
◯ I have been prescribed opioid/narcotic pain medication in the past, but I am not currently using it
◯ I currently use opioid/narcotic pain medication sometimes, but not daily
◯ I am currently using opioid/narcotic pain medication daily

If you have ever been prescribed opioid/narcotic pain medications, complete 3a and 3b. If not, skip to question 4.

3a. Have you ever received information on how to store opioid/narcotic medication? Select all that apply:

☐ Yes, from a pharmacist
☐ Yes, from a healthcare provider
☐ Yes, from another source – please specify: ____________________
☐ No, I have never received such information
☐ I don’t recall

3b. Have you ever received information on how to dispose of opioid/narcotic medication? Select all that apply:

☐ Yes, from a pharmacist
☐ Yes, from a healthcare provider
☐ Yes, from another source – please specify: ____________________
☐ No, I have never received such information
☐ I don’t recall

4. **Do you have any opioid/narcotic medication in your household that is no longer being used or is expired? Select all that apply:**
   - ☐ Yes, opioid/narcotic medication prescribed to me
   - ☐ Yes, opioid/narcotic medication prescribed to someone else
   - ☐ No

5. **How do you store opioid/narcotic medication in your household? Select one:**
   If you do not have any currently, indicate how you would store it if you did.
   - ◯ Cabinet/storage with a latch
   - ◯ Cabinet/storage with a lock
   - ◯ Cabinet/storage with no latch or lock
   - ◯ Other – please specify: ______________________________

6. **How do you dispose of your unused opioid/narcotic pain medication? Select all that apply:**
   If you do not have any currently, indicate how you would dispose of it if you did.
   - ☐ Flush down the sink or toilet
   - ☐ Throw away in garbage
   - ☐ Mix with undesirable material (e.g. kitty litter, coffee grounds) and throw in garbage
   - ☐ Return it to the pharmacy or a community take-back program
   - ☐ I would not dispose of it
   - ☐ Other – please specify: ______________________________

7. **Have you ever shared prescription opioid/narcotic medication with another person? Select all that apply:**
   - ☐ Yes, I have shared opioid/narcotic medication prescribed to me
   - ☐ Yes, I have used opioid/narcotic medication prescribed to someone else
   - ☐ No

8. **Do children, teenagers, or young adults live in your home or visit your home? Select all that apply:**
   - ☐ Yes, young children aged 0-6 years
   - ☐ Yes, older children aged 7-12 years
   - ☐ Yes, teenagers aged 13-17 years
   - ☐ Yes, young adults aged 18-25 years
   - ☐ None of the above

9. **If you had unused opioid/narcotic medication, would you be willing to bring it to your next appointment at the hospital for disposal?**
   ◯ No ◯ Yes

   9a. *If no*, please provide a reason. Select all that apply:
   - ☐ I am afraid my pain will come back and I'm not willing to be without it
   - ☐ If I need it again, I will not be able to get it from a doctor in a timely manner
   - ☐ I do not want to throw it away because I paid for it
   - ☐ My friends or family members may need it
   - ☐ I would prefer to dispose of it myself
   - ☐ Other – please specify: ______________________________

Thank you for taking the time to complete this survey.

Ask your doctor, nurse, or pharmacist for more information on how to safely use and dispose of your medications.
