# Supplementary material for: The potential for diversion of prescribed opioids among orthopaedic patients: Results of an anonymous patient survey
Source: PLoS One. 2021 Aug 26;16(8):e0256741. doi: 10.1371/journal.pone.0256741 (PMC8389484; doi:10.1371/journal.pone.0256741)
Supplement: Attachment — Submitted filename: opioid survey_response to reviewers.docx [file pone.0256741.s003.docx]

**Manuscript PONE-D-21-17272**: The potential for diversion of prescribed opioids among orthopaedic patients: results of an anonymous patient survey

**Response to Reviewers**

Editor comments:

| Comment | Response | Page number(s) |
| --- | --- | --- |
| 1. Please ensure that your manuscript meets PLOS ONE's style requirements, including those for file naming. | Thank you for pointing this out. We have revised our manuscript and supporting files to match PLOS ONE’s specifications. | n/a |
| 2. PLOS requires an ORCID iD for the corresponding author in Editorial Manager on papers submitted after December 6th, 2016. Please ensure that you have an ORCID iD and that it is validated in Editorial Manager. | My ORCID iD has been entered in Editorial Manager. | n/a |
| 3. Please include additional information regarding the survey or questionnaire used in the study and ensure that you have provided sufficient details that others could replicate the analyses. For instance, if you developed a questionnaire as part of this study and it is not under a copyright more restrictive than CC-BY, please include a copy, in both the original language and English, as Supporting Information. | As suggested, we have included the questionnaire as a Supplemental Appendix. | Reference on page 4, line 75 |
| 4. We note that you have indicated that data from this study are available upon request. PLOS only allows data to be available upon request if there are legal or ethical restrictions on sharing data publicly. For information on unacceptable data access restrictions, please see [http://journals.plos.org/ plosone/s/data-availability#loc-unacceptable-data-access-restrictions](http://journals.plos.org/%20plosone/s/data-availability#loc-unacceptable-data-access-restrictions).  In your revised cover letter, please address the following prompts:  a) If there are ethical or legal restrictions on sharing a de-identified data set, please explain them in detail (e.g., data contain potentially identifying or sensitive patient information) and who has imposed them (e.g., an ethics committee). Please also provide contact information for a data access committee, ethics committee, or other institutional body to which data requests may be sent.  b) If there are no restrictions, please upload the minimal anonymized data set necessary to replicate your study findings as either Supporting Information files or to a stable, public repository and provide us with the relevant URLs, DOIs, or accession numbers.  We will update your Data Availability statement on your behalf to reflect the information you provide. | We have addressed this issue in our cover letter: the data underlying the findings of this study contain sensitive patient information and cannot be shared publicly, per our institutional research ethics board (REB). Readers can request access to the data by completing a data request following a research proposal and a data access agreement approved by the University Health Network (UHN) Arthritis Program Research Committee and the UHN REB, signed by all parties. The UHN Arthritis Program Research Committee can be contacted via Christian Veillette at [Christian.Veillette@uhn.ca](mailto:Christian.Veillette@uhn.ca). The UHN REB can be contacted at: University Health Network Research Ethics Board, 700 University Avenue, 4th Floor, Toronto, Ontario M5G 1Z5, Canada (e-mail: [reb@uhnresearch.ca](mailto:reb@uhnresearch.ca), phone: 1-416-581-7849). | n/a |
| 5. Thank you for stating the following in the Competing Interests section:  'I have read the journal's policy and the authors of this manuscript have the following competing interests: Y. Raja Rampersaud has received royalties from Medtronic and holds investments in Arthur Health Corporation. All other co-authors have no competing interests to report.'  Please confirm that this does not alter your adherence to all PLOS ONE policies on sharing data and materials, by including the following statement: "This does not alter our adherence to PLOS ONE policies on sharing data and materials.” (as detailed online in our guide for authors [http://journals.plos.org/plosone/s/competing-interests](about:blank)).  If there are restrictions on sharing of data and/or materials, please state these. Please note that we cannot proceed with consideration of your article until this information has been declared.  Please include your updated Competing Interests statement in your cover letter; we will change the online submission form on your behalf. | I confirm that the competing interests listed do not affect my adherence to PLOS ONE policies on sharing data and materials. As instructed, our cover letter includes our updated Competing Interests statement. | n/a |
| Please review your reference list to ensure that it is complete and correct. If you have cited papers that have been retracted, please include the rationale for doing so in the manuscript text, or remove these references and replace them with relevant current references. Any changes to the reference list should be mentioned in the rebuttal letter that accompanies your revised manuscript. If you need to cite a retracted article, indicate the article’s retracted status in the References list and also include a citation and full reference for the retraction notice. | Thank you for this reminder. We have confirmed that our reference list is correct and that none of the papers cited in our manuscript have been retracted.  We have added one reference (39. Shah et al., 2017) based on Reviewer 2’s comments, noted below. | n/a |

Reviewer 1 comments:

| Comment | Response | Page number(s) |
| --- | --- | --- |
| I think this analysis in your article is an important finding, considering the opioid epidemic in Canada and the numbers you raised for hospitalization.  The abstract clearly summarizes the study and is adequate.  The introduction is adequate. | Thank you! Your thorough review is greatly appreciated. |  |
| For the method part, you write that existing studies defined safe storage location as protected by a lock. So why did you consider both latch and lock as safe for your study? A Latch for me is not really safe, as beginning with small children or anyone else getting to that room will be able to open it. Does your data change when you only consider a lock as a safe storage location? | This is a fair point. We included latched locations as “safe” because locking up opioids is not explicitly recommended in official Canadian guidelines [1]. However, as the reviewer states, both the existing literature and practicality suggest that only a locked location should be considered safe.  Therefore, we have re-analyzed Table 2 and Table 4 with storage methods dichotomized as “locked” (i.e., safe) versus “not locked” (i.e., not safe).  The relationship between children and storage method did not change with the revised definition of safe storage—respondents using safe and unsafe methods reported no difference in rates of minors/young adults in the household. Respondents using non-locked storage locations still frequently reported children (20.6%) or teenagers/young adults (28.8%) in the household.  We have revised our manuscript to reflect this more justifiable definition of safe opioid storage. | Page 2: line 33;  Page 5: line 101-103;  Page 11: Table 2, subheading “Opioid handling behaviors”  Page 13: line 168-169, 176;  Page 14: line 199-204;  Page 15: Table 4, line 217  Page 17: line 249-250 |
| What would be interesting is establishing a education program e.g. in your clinic and to the measurement again, to see if your hypothesis that education in safe storage and so on has any effect on return rates or safe storage. In the same way it might be interesting to investigate, as you say, if patient handling with opioids has changed by the awareness of the opiod epidemic und media attention. | We agree completely—our subsequent work will aim to determine whether an education program is effective for promoting safer opioid handling among patients. We have included this in our Discussion. | Page 17: line 259-261 |
| You state that patients with unused opioids at home have significantly less likely received storage information or disposal instructions. The questions that raises to me is on the other hand is if patients who have received storage information and disposal instructions are more likely to use a safe storage location, have higher rates in returning opioids, have less unused opioids at home and so on. This is the questions to answer if one wants to see if education on opiod use has any effect. This is somehow the conclusion of your study, that more education is needed, but is there any proof that education changes the rates of safe opiod handling? Can you answer that out of your data? | Thank you for raising this insightful question. We do have the data to compare opioid handling behaviors in respondents who received storage and disposal instructions versus respondents who did not receive these instructions.  Respondents in our sample who received storage and disposal instructions from a pharmacist or health care provider did report safer handling behaviors: they were less likely to have unused opioids at home (p=0.005), more likely to keep opioids in a locked location (p<0.001), and more likely to dispose of opioids at a pharmacy or take-back program (p<0.001).  We have revised the manuscript to report these results in the Results and Discussion sections, and the full comparative analysis results are included in S1 Table. | Page 2, lines 36-37, 40-41;  Page 13, line 179-184;  Page 17: line 254 |

Reviewer 2 comments:

| Comment | Response | Page number(s) |
| --- | --- | --- |
| Abstract/ Results: consider adding a denomination, e.g. '(past) prescription opioid use' to better describe the study population in summary. | We have clarified this in the abstract methods and results. | Page 2: lines 26, 30 |
| Table 2 is an extensive table, leading to reduced legibility. Please provide some separation between parts of the table, either within this table, or by creating multiple tables. | Thank you for pointing this out. We have reformatted Table 2 with sub-headings. | Pg. 10-12 |
| Discussion, second paragraph: limitations were described in profound detail. However, it could be of interest to add some background information on chronic opioid use in relation to the study population. E.g.: J Pain. 2017 November ; 18(11): 1374–1383 | The cited paper is a good discussion of risk factors for long-term opioid use; we have included it as supporting evidence of the concurrent need to improve opioid prescribing practices in our Discussion. (reference 39: Shah et al., 2017) | Pg. 16, line 238 |

**References**

1.	Government of Canada. About Opioids. Ottawa, ON; 2019. Available: https://www.canada.ca/en/health-canada/services/substance-use/problematic-prescription-drug-use/opioids/about.html
